# EMPOWERING OLDER ADULTS THROUGH SMART TECHNOLOGY: A NEW ERA IN AGING-IN-PLACE AND HOME HEALTH CARE

**DOI:** 10.1093/geroni/igae098.2024

**Published:** 2024-12-31

**Authors:** Daejin Kim

**Affiliations:** Florida State University, TALLAHASSEE, Florida, United States

## Abstract

Demographic aging presents unique global challenges, especially regarding the preference of many older adults to age in place. Traditional physical environments often inadequately support the complex needs of this demographic, highlighting the need for innovative approaches. This presentation focuses on the integration of smart home technologies that are powered by machine-learning algorithms to facilitate successful aging in place. Smart home technologies encompass an array of sensors, devices, and machine-learning analysis, and can significantly enhance the wellness and independence for older adults through home automation, health monitoring, and daily activity tracking through machine learning algorithms. They provide essential insights into the functional, cognitive, and social well-being of older individuals, thus supporting their independence and enriching their quality of life. Despite their immense potential, a knowledge gap persists regarding the availability and integration of smart home technologies in homes designed for older adults. This presentation addresses this gap by highlighting the practical applications of these technologies for healthcare professionals, architects, and policy makers. It discusses challenges like privacy, user-friendliness, and affordability, and explores future advancements in this field. The presentation also emphasizes the importance of adopting technological solutions to transform built environments for older adults through a comprehensive overview of the critical role of smart home technologies. It underscores how tailored, effective technological support can significantly improve living conditions for older adults, enabling them to live safely and independently in their preferred environment. The presentation is a call to action for creating more supportive and adaptable living spaces for our aging society.

